# Phenotypic plasticity alone cannot explain climate-induced change in avian migration timing

**DOI:** 10.1002/ece3.367

**Published:** 2012-08-29

**Authors:** Josh Buskirk, Robert S Mulvihill, Robert C Leberman

**Affiliations:** 1 Institute of Evolutionary Biology and Environmental Studies, University of ZürichCH-8057, Zürich, Switzerland; 2 Powdermill Avian Research Center, Carnegie Museum of Natural HistoryRector, Pennsylvania, 15677-9605; 3 National AviaryPittsburgh, Pennsylvania, 15212

**Keywords:** Bird migration, evolution, phenology, phenotypic plasticity, temperature

## Abstract

Recent climate change has been linked to shifts in the timing of life-cycle events in many organisms, but there is debate over the degree to which phenological changes are caused by evolved genetic responses of populations or by phenotypic plasticity of individuals. We estimated plasticity of spring arrival date in 27 species of bird that breed in the vicinity of an observatory in eastern North America. For 2441 individuals detected in multiple years, arrival occurred earlier during warm years, especially in species that migrate short distances. Phenotypic plasticity averaged −0.93 days °C^−1^ ± 0.70 (95% CI). However, plasticity accounted for only 13–25% of the climate-induced trend in phenology observed over 46 years. Although our approach probably underestimates the full scope of plasticity, the data suggest that part of the response to environmental change has been caused by microevolution. The estimated evolutionary rates are plausible (0.016 haldanes).

## Introduction

Phenological responses to climate change are well-documented in plants and animals (Parmesan and Yohe 2003; Root et al. 2005; Menzel et al. 2006; Cleland et al. 2007; Phillimore et al. 2010). It is widely assumed that much of the shift in phenology is due to facultative changes in the activities or physiologies of individuals induced by environmental conditions, known as phenotypic plasticity (Both and Visser 2001; Hüppop and Hüppop 2003; Gienapp et al. 2008; Van Buskirk 2012). This assumption is justified by everyday observations of individual responses to short-term fluctuations in weather, such as accelerated bud-burst in long-lived trees during warm spring weather. Indeed, data from individuals tracked over multiple years in longitudinal studies have revealed that plasticity induced by weather can sometimes explain most of the observed change in phenology (Réale et al. 2003; Charmantier et al. 2008; Valtonen et al. 2011) and other traits (Teplitsky et al. 2008; Ozgul et al. 2010).

However, phenotypic plasticity is not the only mechanism that can produce population responses to climate change. Gradual or sudden shifts in the selection regime can be triggered by environmental change, and these in turn can alter the genetic composition of populations. Indeed, rapid evolved responses to climate change are widely anticipated by evolutionary biologists (Bradshaw and Holzapfel 2001; Davis et al. 2005; Gienapp et al. 2008; Hoffmann and Willi 2008; Hoffmann and Sgro 2011), and already have been observed in a few cases (Umina et al. 2005; Bradshaw and Holzapfel 2008). Evolved and plastic responses may appear similar to an observer, because both cause phenotypic shifts in an adaptive direction. Data are rarely available to differentiate between the two mechanisms, because it is challenging to estimate plasticity and evolution in wild populations that are not amenable to experimentation.

In this study, we adopted an indirect approach to detect microevolutionary change in the phenology of 27 species of bird in eastern North America. First, we estimated the magnitude of temperature-induced phenotypic plasticity in spring arrival date by recording the effects of annual variation in spring temperature on the behavior of thousands of individuals. Our main question was whether phenotypic plasticity alone can explain observed shifts in migration phenology between 1961 and 2006. If not, the shift that remained unaccounted for was considered at least partly due to microevolutionary change in migratory behavior. Finally, we asked whether the putative microevolutionary change was within the range of plausible evolutionary rates, given what is known about the genetic basis of avian phenology.

## Methods

### Study area

Between June 1961 and August 2006, we operated about 35 mist nets for 5–6 days each week on a 10-ha study area at Powdermill Nature Reserve (PNR), a field station maintained by Carnegie Museum of Natural History in Pennsylvania, USA (elevation 400 m; 40.163°N, 79.267°W). Ringing methods and net locations remained largely unchanged during this study, and most birds were processed by just two people (R. C. Leberman and R. S. Mulvihill). Detailed field methods are in Marra et al. (2005) and Van Buskirk et al. (2009).

### Temperature-induced phenotypic plasticity

We estimated the phenological response to temperature variation of individual birds that were captured as adults in at least two different years. This included all species that breed within the study area except for those that overwinter locally or those with <15 individuals recorded. Arrival was defined as the first date on which the individual was captured in spring or early summer. Temperature was measured over a geographic region extending 1200 km south of PNR. We averaged data from three randomly selected weather stations in the United States Historical Climatology Network (USHCN; Williams et al. 2007) from each of the nine states to the south of our study area. This represents the area through which birds migrate to reach PNR or within which short-distance migrants spend the winter. We also used temperature data from USHCN weather stations within 200 km of PNR, but found only weak plasticity induced by temperature at this local spatial scale (data not shown).

Temperature-induced phenotypic plasticity was the slope of the regression of arrival date against temperature, estimated from a mixed-effects linear model (“random regression”; Nussey et al. 2007; Brommer et al. 2012). Fixed effects were the age of the bird in years, temperature, migration distance, and the interaction between temperature and migration distance. Random effects were species (*N* = 27), individuals within species (*N* = 2441), and terms that estimated heterogeneity in slopes of species and individuals against temperature. The total sample size was 5988 observations. Age was included because many passerines migrate earlier as they become older (Stewart et al. 2002). Age was not known for some individuals, so we assumed that these were 1-year old on the first year of capture. If adult survival is as high as 50%, this assumption would be correct for half the individuals of unknown age. Mistaken age assignment will have no influence if the relationship between arrival and age is approximately linear, for which there is some evidence (Morton and Derrickson 1990). Migration distance was included because the phenological response to climate change is known to be stronger in short-distance migrants (Lehikoinen and Sparks 2010), possibly because they have more opportunity to express facultative responses to spring weather conditions. Species that overwinter in the southern United States were considered short-distance migrants, whereas those that overwinter primarily south of North America were long-distance migrants (see Table A1; Poole 2008).

The time of year during which temperature influences migratory behavior most strongly is not known, so we calculated mean temperatures for 127 time intervals and fitted the model described above for every interval (see Husby et al. 2010). Starting dates began on 20 January and occurred at 5-day intervals thereafter. The final dates for time intervals were at least 20 days after the starting date and also occurred at 5-day intervals up to 90 days. Intervals that extended beyond 1 June were not considered. Temperature-induced plasticity in arrival date was taken from the time interval giving the most significant slope of arrival date against temperature. Because species may differ in the time interval to which they are most sensitive, we also performed separate regressions for each species over the 127 time intervals, and again recorded plasticity from the time interval with the most significant slope. Analyses were implemented with the lme4 package in R version 2.13.2 (Baayen et al. 2008).

### Changes due to plasticity and microevolution

We compared the observed change in phenology over 46 years with the magnitude of plasticity projected over the same time period. The estimate of plasticity from the hierarchical model described above, in units of days °C^−1^, was multiplied by the trend in mean spring temperature between 1961 and 2006 from the same time interval that yielded maximal plasticity. This gave an estimate of the change in phenology due purely to plasticity, in days year^−1^. The observed change in arrival time came from records of first capture dates for locally breeding individuals of the same 27 species. A bird was judged to be a local breeder either if it was recaptured over a time period of ≥30 days within a single breeding season, or if it was captured as an adult in multiple years. Migrants are virtually never caught in more than 1 year, nor do they remain on our study area for many weeks during summer.

To evaluate the plausibility of microevolution as an explanation for change in phenology, we calculated the rate of evolutionary change that would be required to produce the observed shift in migration timing, after removing change due to plasticity. The measurement unit we used, the haldane, is the change in standard deviation (SD) units of the trait per generation (Gingerich 1993). Phenotypic SD in first arrival date was calculated for locally breeding individuals, separately for each species and year, and then averaged across years. Generation time was calculated as *α* + (*s*/(1−*s*), where *α* is the age at first reproduction and *s* is the annual survival rate (Lande et al. 2003). Age at first reproduction is 1 year for the species in this study, and estimates of *s* came from Martin and Li (1992) and the MAPS database maintained by the Institute for Bird Populations (http://www.birdpop.org/). We estimated selection differentials required to produce the observed microevolutionary change, using the breeder's equation, by dividing haldanes by the heritability in phenology. Heritabilities spanned the range in the literature for field estimates of passerine arrival date and egg-laying date: 0.19 and 0.54 (Potti 1998; Møller 2001; Sheldon et al. 2003; Pulido 2007).

## Results

**Figure 1:**
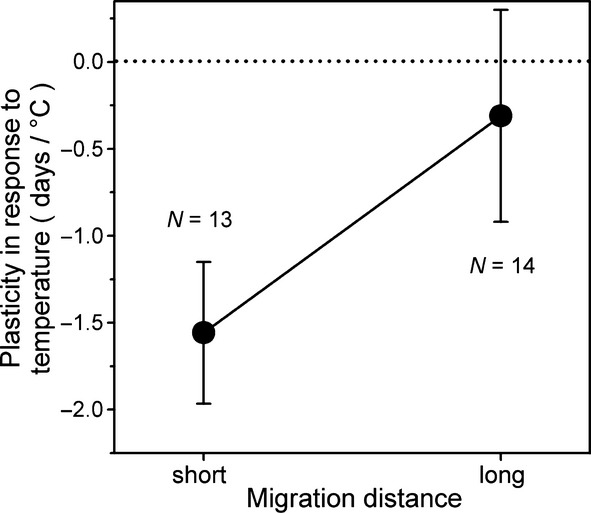
Temperature-induced plasticity in spring arrival date of birds at Powdermill Nature Reserve in western Pennsylvania, USA. Values are estimates of plasticity (±1 SE) from a mixed-effects linear model regressing arrival date against temperature. Temperature is averaged over a large region extending 1200 km to the south of the study area. Sample sizes are the number of species. Individuals of species that overwinter in North America reacted more strongly to warm years, as reflected in the interaction between temperature and migration distance (Table 1).

Significant phenotypic plasticity was indicated by earlier arrival dates during warm years for individual birds detected as adults in multiple years of the study. The time interval that produced the strongest temperature-induced plasticity was a broad period between 1 March and 20 May. The average magnitude of plasticity, estimated at the level of species in a hierarchical mixed-effects linear model, was −0.931 ± 0.698 (95% CI) days °C^−1^ (*P* = 0.0090) That is, individual birds arrived on their breeding area nearly 1 day earlier for every one-degree increase in temperature in southeastern North America (Fig. 1).

The response to temperature was stronger in short-distance migrants, which spend the winter in the southern United States, than in long-distance migrants, which spend the winter primarily to the south of North America (Fig. 1; migration distance-by-temperature interaction in Table 1). This supports the notion that impacts of climate change on the phenology of short-distance migrants are greater in part because these species display greater plasticity (Lehikoinen et al. 2004). Birds returned earlier to their breeding territories as they grew older, by about 1.7 days year^−1^. Random effects in Table 1 highlight variation in arrival dates of species and individuals, but there was no evidence for heterogeneity in temperature-induced plasticity among species or individuals.

**Table 1:** Mixed-effect linear models estimating temperature-induced plasticity in arrival date of birds sampled at Powdermill Nature Reserve in western Pennsylvania, USA. The response variable is arrival date. The table reports coefficients for fixed effects and variance components for random effects. Boldface highlights estimates that were significant. Arrival date was measured in days, age in years, and temperature in °C. The range of dates over which temperature was averaged was 1 March until 20 May. Individual was nested within species. Sample size was 5988 observations from 2441 individuals of 27 species

Source	Level	Estimate	SE	*P*-value
Fixed effects (coefficients)				
Age		−1.721	0.162	0.0001
Migration distance	Long	**22.712**	**3.790**	**0.0001**
Temperature		−**1.558**	**0.407**	**0.0001**
Migr dist × temperature	Long	**1.258**	**0.633**	**0.0469**
Random effects (variance components)				
Species		**92.656**	**.**	**0.0001**
Species × temperature		0	.	.
Individual		**38.913**	**.**	**0.0001**
Individual × temperature		0	.	

**Figure 2:**
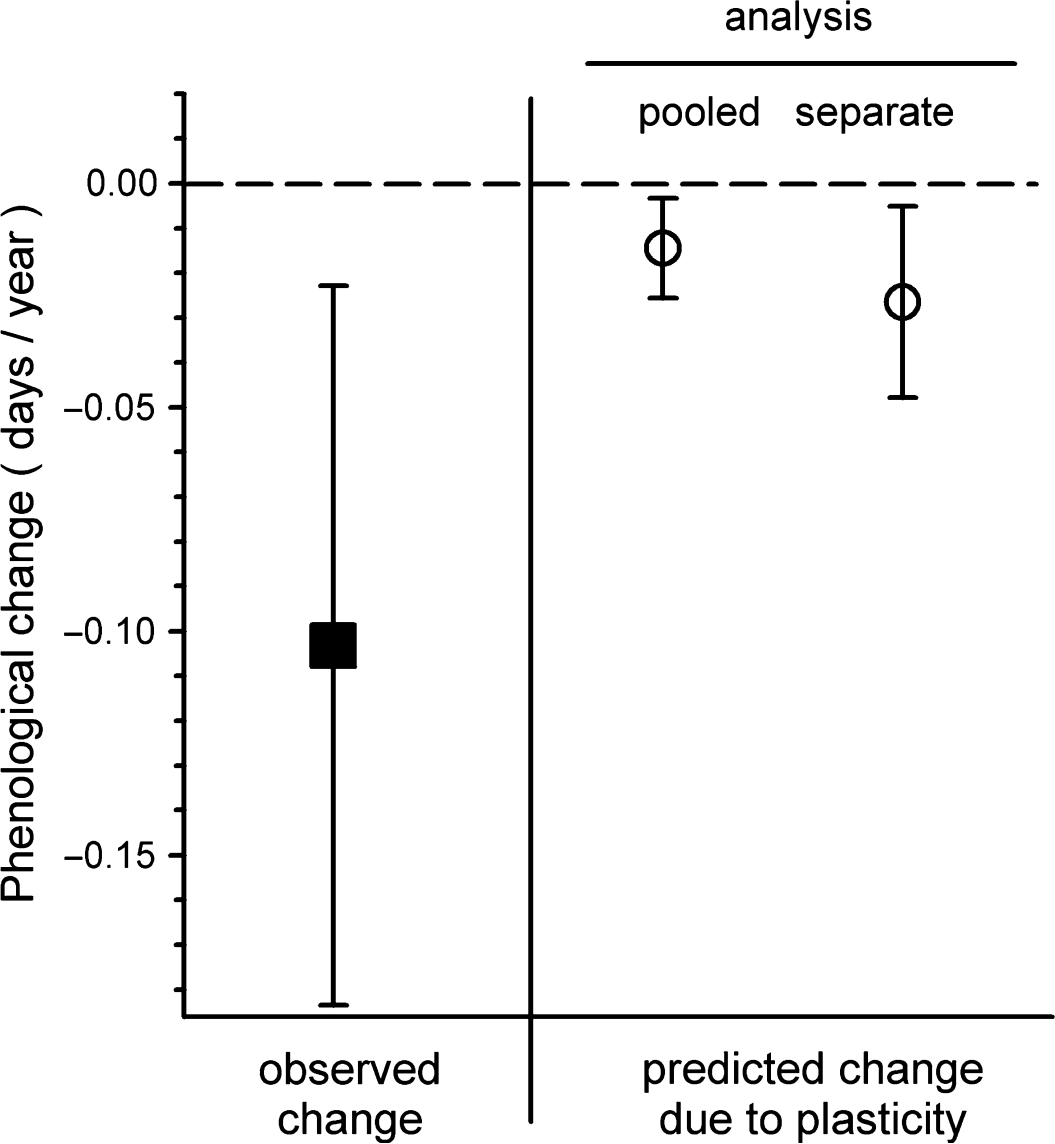
Rate of change in the date of spring arrival for 27 bird species at Powdermill Nature Reserve between 1961 and 2006 (filled square), and the rate of change expected if the entire response arose from individual-level plasticity induced by warming temperatures (open circles). Plasticity was estimated from a single hierarchical mixed-effect model conducted on the entire dataset (“pooled”), and from separate models for each species (“separate”). Temperature was averaged over southeastern North America. Error bars represent ± 95% CI.

Was phenotypic plasticity sufficient to explain the shift in migration timing observed over the years? Locally breeding adults of the 27 species studied here have been returning earlier to PNR since the early 1960s by an average of 0.103 ± 0.080 days year^−1^ (mean ± 95% CI). The change in phenology predicted under a model of pure phenotypic plasticity fell far short of the change in arrival date that we observed (“pooled” analysis in Fig. 2). Spring temperatures in southeastern North America have increased at the rate of 0.0156 °C year^−1^; this translates to a predicted plastic response of −0.0145 days year^−1^, which is 13.4% ± 10.3 (95% CI) of observed phenological change.

Estimates of phenotypic plasticity from separate analyses for each species were somewhat larger than that in the pooled analysis shown in Table 1, probably because the different species responded to different temperature intervals (Fig. 2). Temperature-induced plasticity averaged −1.704 ± 1.350 (95% CI) days °C^−1^, which translates to a predicted plastic response of −0.0265 days year^−1^ (24.6% ± 19.9 of observed change). Results for each species are in Table A1.

These findings imply a modest rate of microevolutionary change. For regional temperature, the shift toward earlier arrival date that was not accounted for by plasticity requires a rate of 0.016 haldanes (phenotypic SD units·generation^−1^). Selection differentials that would cause this evolutionary rate are between 0.029 and 0.084 SD units, for heritabilities of 0.54 and 0.19, respectively (Møller 2001; Sheldon et al. 2003). Table A1 lists estimates for each species separately.

## Discussion

These results suggest that birds may be adjusting to climate change with a combination of phenotypic plasticity and rapid microevolution. Biologists have expected that evolution will be an important ingredient of climate change adaptation, but it has been difficult to differentiate the relative contributions of evolved and environmentally induced change (Gienapp et al. 2008; Hoffmann and Willi 2008; Hoffmann and Sgro 2011). In our study, temperature-induced plasticity was quantitatively important and is likely to be adaptive because it allows individuals to match their activities (e.g., migration, nesting) with the timing of other biotic events in the environment (e.g., bud-burst, insect emergence) (Dunn et al. 2011). The share of phenological change that was not accounted for by plasticity may have been caused − at least in part − by a genetic response to natural selection. Selection favoring earlier reproduction is known to occur in bird populations during warm years (Van Noordwijk et al. 1995; Both and Visser 2001; Charmantier et al. 2008). Microevolutionary response to selection is plausible in this case because the 46-year duration of the study spans at least 20 generations, and the timing of avian migration and reproduction have reasonably high heritabilities (Sheldon et al. 2003; Pulido 2007). Moreover, the rate of genetic evolution that we calculated, about 0.016 haldanes, is lower than 26% of the 2420 published estimates of evolutionary rates compiled by Hendry et al. (2008). The strength of selection required to sustain this rate of evolution is not exceptionally high. Depending on assumptions about the heritability of migration timing, between 61% and 85% of directional selection gradients compiled in Kingsolver and Diamond's (2011) database are larger than the coefficients that we estimated. Of course, these calculations assume continuous directional selection over 46 years; inconsistent selection imposed only during warmer years would entail greater selection coefficients and rates of response.

At face value, these results suggest that the majority of phenological change observed at PNR reflects microevolution. However, this conclusion is based on indirect evidence and relies on at least two important assumptions. The first is that plasticity is triggered by variation in temperature rather than some other feature of the environment that signals whether the season is early or late. The second is that migratory behavior is sensitive to temperature averaged over a large region to the south of our study area. Violation of either assumption could lead to an under-estimation of phenotypic plasticity. That is, estimates of plasticity might be higher if we knew either the climatic features to which birds pay attention or the exact migratory route they follow before reaching PNR. There is evidence supporting the importance of temperature – or a factor closely correlated with temperature – in dictating spring migration. Many studies observe a strong association between temperature and annual variation in spring arrival, even after accounting for long-term trends (Van Buskirk et al. 2009; Lehikoinen and Sparks 2010; Knudsen et al. 2011). Rates of movement during spring migration have been tied to temperature in some studies (Both et al. 2005; Marra et al. 2005; Tottrup et al. 2010). In a few species, temperature is thought to induce the phenology of egg-laying of individual birds (Both and Visser 2001; Charmantier et al. 2008). Of course, several factors beyond temperature are also known to be important (e.g., Berthold 1996; Hüppop and Hüppop 2003; Knudsen et al. 2011). The region of study is justified by information on spring migratory routes in eastern North America, although the exact paths followed by each species are not well enough known to incorporate into our analyses (Poole 2008). In summary, we suspect that neither assumption is entirely correct, and as a consequence the true scope of phenotypic plasticity is somewhat higher – and the extent of microevolutionary change is lower – than the estimates presented here. Could violations of these assumptions have caused a four to sevenfold underestimate of plasticity, as would be required to fully explain the observed phenological change since 1961 (Fig. 2)? We do not know. However, we believe our findings are sufficiently strong to justify seriously considering a role for microevolution in the phenological responses of birds to climate change.

The relative magnitudes of plasticity and genetic adaptation are important for understanding limits of biotic responses to ongoing environmental change. If organisms are primarily exhibiting phenotypic plasticity, as has been widely expected (Both and Visser 2001; Hüppop and Hüppop 2003; Menzel et al. 2006; Gienapp et al. 2008; Knudsen et al. 2011), then the costs and limits of plasticity are relevant (DeWitt et al. 1998; Van Buskirk and Steiner 2009) along with conditions promoting the evolution of further adaptive plasticity (Bradshaw 1965). But if microevolution contributes to observed responses, then we should focus on key factors that limit adaptation (Barton and Partridge 2000; Willi et al. 2006; Hoffmann and Sgro 2011). In this case, the pace of evolution relative to the rate of environmental change will be important (Chevin et al. 2010) as well as the possibility that evolved responses can soon become maladaptive in fluctuating environments (Van Buskirk 2012).

Indirect evidence suggests that responses to climate change may already be constrained by limits to adaptation. Møller et al. (2008) and Saino et al. (2011) report that European bird species that have experienced the steepest population declines have also shown the smallest advancements in the timing of spring migration in recent decades. Considering the possible importance of microevolution, the causes of this pattern may include evolutionary limits associated with small population size. If population declines have impacted genetic effective population sizes, then genetic drift will diminish the effectiveness of selection on phenology (Slatkin 1985) and genetic erosion may compromise the capacity of smaller populations to respond to selection (Willi et al. 2006). Moreover, selection for early reproduction may be weakened in the first place, if declining species experience a reduction in local breeding density (Ahola et al. 2009). This example illustrates how appreciation of the population-level consequences of recent environmental change can be guided by information about mechanisms of climate adaptation.

If phenological responses to environmental change arise from multiple causes, this would be encouraging for the prospects of migratory birds in the short-term. Rapid genetic response to climate change is widely seen as a critical component of the kind of adaptation that will be required of many organisms (Davis et al. 2005; Hoffmann and Sgro 2011). At the same time, the contribution of plasticity will allow individuals to adjust their phenotype to short-term environmental fluctuations, which are projected to increase under most scenarios of climate change.

## Appendix

**Table A1:** List of species included in this study and summary of the main results for each species. Migration distances come from Poole (2008). The observed phenological change is the slope of first capture date regressed against year, including only locally breeding adults (days year^−1^). The number of individuals is the number of adult birds for which estimates of temperature-induced plasticity were available. Estimated plasticity is the change in arrival time of individual birds for each 1°C change in mean temperature over the time interval specified, estimated from separate analyses of each species. Annual adult survival estimates are from Martin and Li (1992) indicated with ^A^, or from the MAPS database indicated with ^M^ (http://www.birdpop.org/nbii/nbiihome.asp). Generation time is 1 + (*s*/(1−*s*), where *s* is annual survival (Lande et al. 2003; Stochastic population dynamics in ecology and conservation). Haldanes is the change in phenology not explained by plasticity induced by regional temperature change (SD units·generation^−1^). Directional selection differentials necessary to produce the observed microevolutionary response were calculated under two assumptions about heritability (*h*^2^ = 0.19, *h*^2^ = 0.54) (Møller 2001; Sheldon et al. 2003)

				Estimated plasticity						
								
Species		Migr. distance	Observed phenol. change	No. indiv.	Interval	Days °C^−1^	Survival	Generation time	Haldanes	Selection differentials
Ruby-throated Hummingbird	*Archilochus colubris*	Long	-0.083	41	6 Mar–25 Apr	−3.38	.	.	.	.
Eastern Phoebe	*Sayornis phoebe*	Short	-6.71	16	20 Jan–19 Feb	−0.014	.	.	.	.
White-eyed Vireo	*Vireo griseus*	Short	-1.41	22	15 Jan–4 Feb	0.007	0.492^M^	1.97	0.007	0.037, 0.013
Red-eyed	*Vireo olivaceus*	Long	1.16	189	21 Mar–10 Apr	0.022	0.553^A^	2.24	0.008	0.042, 0.015
Tree Swallow	*Tachycineta bicolor*	Short	5.05	20	4 Feb–6 Mar	−0.878	0.386^M^	1.63	-0.172	-0.905, -0.319
House Wren	*Troglodytes aedon*	Short	2.51	30	11 Mar–31 Mar	−0.074	0.286^A^	1.40	-0.007	-0.037, -0.013
Eastern Bluebird	*Sialia sialis*	Short	-6.41	18	20 Jan–9 Feb	−0.512	0.490^A^	1.96	-0.071	-0.374, -0.131
Wood Thrush	*Hylocichla mustelina*	Long	-6.88	30	4 Feb–5 Apr	−0.011	0.628^A^	2.69	-0.001	-0.005, -0.002
American Robin	*Turdus migratorius*	Short	-3.99	68	1 Mar–20 Apr	−0.168	0.600^A^	2.50	-0.019	-0.100, -0.035
Gray Catbird	*Dumetella carolinensis*	Long	1.58	149	6 Mar–26 Mar	−0.119	0.577^A^	2.36	-0.027	-0.142, -0.050
Golden-winged Warbler	*Vermivora chrysoptera*	Long	-1.43	45	20 Apr–10 May	−0.142	.	.	.	.
Yellow Warbler	*Dendroica petechia*	Long	0.570	101	9 Feb–1 Mar	0.013	0.512^A^	2.05	0.004	0.021, 0.007
American Redstart	*Setophaga ruticilla*	Long	3.66	37	20 Jan–9 Feb	0.140	0.670^A^	3.03	0.042	0.221, 0.078
Ovenbird	*Seiurus aurocapilla*	Long	-5.83	15	16 Mar–25 Apr	−0.044	0.638^A^	2.76	-0.010	-0.053, -0.019
Louisiana Waterthrush	*Seiurus motacilla*	Long	-7.36	49	16 Mar–5 Apr	−0.056	0.364^M^	1.57	-0.006	-0.032, -0.011
Common Yellowthroat	*Geothlypis trichas*	Long	-2.75	166	5 Apr–25 May	−0.148	0.542^A^	2.18	-0.033	-0.174, -0.061
Hooded Warbler	*Wilsonia citrina*	Long	5.37	15	16 Mar–15 Apr	−0.123	0.438^M^	1.78	-0.021	-0.111, -0.039
Yellow-breasted Chat	*Icteria virens*	Long	0.109	26	20 Apr–20 May	−2.55	0.373^M^	1.59	0.031	0.163, 0.057
Eastern Towhee	*Pipilo erythrophthalmus*	Short	-0.138	45	5 Apr–25 Apr	2.57	0.556^A^	2.25	-0.020	-0.105, -0.037
Chipping Sparrow	*Spizella passerina*	Short	-0.126	151	21 Mar–20 Apr	−0.96	0.480^M^	1.92	-0.018	-0.095, -0.033
Field Sparrow	*Spizella pusilla*	Short	0.115	132	11 Mar–10 Apr	−2.26	0.460^A^	1.85	0.019	0.010, 0.035
Song Sparrow	*Melospiza melodia*	Short	0.061	123	24 Feb–5 Apr	−2.21	0.556^A^	2.25	0.008	0.042, 0.015
Indigo Bunting	*Passerina cyanea*	Long	-0.135	137	15 Apr–5 May	−1.32	0.476^A^	1.91	-0.032	-0.168, -0.059
Red-winged Blackbird	*Agelaius phoeniceus*	Short	-0.449	86	19 Feb–11 Mar	−2.54	0.530^A^	2.13	-0.059	-0.311, -0.109
Brown-headed Cowbird	*Molothrus ater*	Short	-0.093	688	6 Mar–25 May	−3.06	0.451^M^	1.82	-0.010	-0.053, -0.019
Baltimore Oriole	*Icterus galbula*	Long	0.015	20	4 Feb–24 Feb	−2.38	0.464^M^	1.87	0.007	0.037, 0.013
Purple Finch	*Carpodacus purpureus*	Short	0.045	22	19 Feb–11 Mar	−5.03	0.426^M^	1.74	0.008	0.042, 0.015
